# Identification of a TRBD zinc finger-interacting protein in *Giardia duodenalis* and its regulation of telomerase

**DOI:** 10.1186/s13071-019-3821-0

**Published:** 2019-11-29

**Authors:** Jing-Tong Zheng, Nan Zhang, Yan-Hui Yu, Peng-Tao Gong, Xian-He Li, Na Wu, Can Wang, Xiao-Cen Wang, Xin Li, Jian-Hua Li, Xi-Chen Zhang

**Affiliations:** 1 Key Laboratory of Zoonosis Research, Ministry of Education, College of Veterinary Medicine, Institute of Zoonosis, Jilin University, Changchun, 130062 China; 2 Department of Pathogenobiology, College of Basic Medicine, Jilin University, Changchun, 130021 Jilin China; 3 State and Local Joint Engineering Laboratory for Animal Models of Human Diseases, Academy of Translational Medicine, First Hospital, Jilin University, Changchun, 130021 China; 4 Clinical Laboratory of Second Hospital, Jilin University, Changchun, 130021 China

**Keywords:** *Giardia duodenalis*, Telomerase, Protein–protein interaction

## Abstract

**Background:**

*Giardia duodenalis* causes giardiasis, with diarrhea as the primary symptom. The trophozoite proliferation of this zoonotic parasite is mainly affected by telomerase, although the mechanism of telomerase regulation has not been thoroughly analyzed.

**Methods:**

This study was performed to identify the telomerase RNA-binding domain (TRBD)-interacting protein in *G. duodenalis* and its regulation of telomerase. Interaction between TRBD and interacting proteins was verified *via* pulldown assays and co-immunoprecipitation (co-IP) techniques, and the subcellular localization of the protein interactions was determined *in vivo via* split SNAP-tag labeling. The hammerhead ribozyme was designed to deplete the mRNA of TRBD-interacting proteins.

**Results:**

Using TRBD as bait, we identified zinc-finger domain (ZFD)-containing proteins and verified it *via* pulldown and co-IP experiments. Protein-protein interaction occurred in the nuclei of 293T cells and both nuclei of *G. duodenalis*. The hammerhead ribozyme depleted ZFD mRNA levels, which reduced the reproduction rate of *G. duodenalis*, telomerase activity and telomere length.

**Conclusions:**

Our findings suggest that ZFD may regulate telomere function in *G. duodenalis* nuclei.

## Background

Telomeres are specialized nucleoprotein complexes at the end of a linear chromosome in eukaryotic cells [1–3] and are maintained by telomerase, an enzyme that is responsible for telomere extension and adds repeated sequences to telomeric ends in cells [4]. Telomerase complexes generally contain three common subunits: telomerase reverse transcriptase (TERT), telomerase RNA (TER), and proteins that bind RNA or are involved in the stabilization and maturation of telomerase complexes. TERT is the catalytic component of telomerase that monitors cellular changes, restores telomerase activity and maintains telomeres [5–7]. TERT can be divided into four main consecutive structural domains: the telomerase essential N-terminus (TEN) domain, the telomerase RNA-binding domain (TRBD), the reverse transcriptase (RT) domain and the C-terminal extension (CTE) domain. The TRBD is responsible for telomerase ribonucleoprotein assembly and is the key component of TERT [8].

Based on in-depth analyses of telomerases, various telomerase-associated proteins have been reported to play important roles in regulating telomerase activity. Studies have shown that hTERT interacts with TPP1 to protect chromosome ends in tumor cells [9]. Additionally, an hTERT fragment (567–594 aa) participates in regulating telomerase activity by interacting with DDRGK1. Moreover, two telomerase proteins from *Tetrahymena thermophila*, p80 and p95, were identified on the basis of their association with telomerase activity and TER [10]. Although the role of telomere-associated proteins in regulating telomerase has been demonstrated, related mechanisms are less well understood.

*Giardia* spp. are zoonotic parasites that greatly impact the health of humans and other mammals [11–13]. *Giardia duodenalis* is a parasite of the duodenum and jejunum that causes giardiasis, with diarrhea being the major symptom. Because *G. duodenalis* is a kind of model organism with a highly reduced genome, it is important to understand complex biological processes in eukaryotic cells. The endomembrane system of *G. duodenalis* is simple and contains only the endoplasmic reticulum (ER) and peripheral vesicles (PVs) [14]. Because *G. duodenalis* can be cultured *in vitro*, it is more amenable for studying such organisms and related diseases than other parasites. At present, the control of giardiasis mainly depends on anti-*Giardia* drugs, such as metronidazole, tinidazole and albendazole. However, since *G. duodenalis* proliferation appears “immortalized” and because a detailed understanding of the molecular biological characteristics of *Giardia* and its regulatory mechanisms is lacking, there is no commercialized vaccine can be used clinically to control giardiasis [15–19]. Thus, the molecular biological characteristics of *G. duodenalis* must be examined, and the nucleus is a key topic in such experiments.

The complete TERT sequence (GenBank: AF195121.1) and repeat sequence (TAGGG)n in *G. duodenalis* have been identified [20–22], and the TRBD and RT of TERT (cl13446 and cd01648) have been found using tools from NCBI. Nonetheless, only a few studies thus far have analyzed TERT localization, telomere length and telomere activity in *G. duodenalis* [23]. Therefore, identifying TERT-associated proteins will complement the available data on the regulatory network of *G. duodenalis* and may contribute to explaining the mechanisms underlying *G. duodenalis* immortalization.

In *G. duodenalis*, daughter cells inherit copies of parental nuclei, but there is little evidence defining the position of the TRBD or telomerase-interacting proteins in *G. duodenalis* nuclei or for whether protein-protein interactions (PPIs) occur in both nuclei or in only one nucleus [18]. Therefore, after identifying TRBD-associated proteins by yeast double-hybrid screening and pulldown and co-immunoprecipitation (co-IP) assays, we explored the location of the TRBD and its associated proteins. Moreover, to ensure the function of TRBD-associated proteins in regulating telomerase, the viral vector-mediated hammerhead ribozyme was used to evaluate telomere length and telomerase activity.

## Methods

### Parasites, yeast strains, and cell culture

The *G. duodenalis* wildtype strain isolated from a dog was genotyped as assemblage A in Changchun, China. Yeast strains Y187 and AH109 were grown at 30 °C in yeast extract peptone dextrose agar (YPDA) medium (LABest, Beijing, China). The 293T cell line was cultured in 1640 medium supplemented with 10% FBS in 5% CO_2_ at 37 °C. To construct a *G. duodenalis* cDNA library, total RNA was extracted from *G. duodenalis* (1 × 10^8^/ml) using TRIzol reagent (Roche, Basel, Switzerland), and first and double-strand cDNAs were synthesized according to the SMART^TM^ cDNA Library Construction kit protocol (Clontech, Palo Alto, USA). The first-strand cDNA sample was amplified using long-distance PCR (LD-PCR), and double-stranded cDNA was purified with CHROMA SPIN+TE 400 columns (Clontech, Saint-Germain-en-Laye, France). Giardivirus vector pC631 was a gift from Wang CC (Department of Pharmaceutical Chemistry, University of California, San Francisco, CA, USA).

### Construction of the *G. duodenalis* cDNA library and yeast two-hybrid screening

A full-length, normalized *G. duodenalis* cDNA library was introduced into the pGADT7-Rec vector and used as prey, as previously described [24]. The prey, which contains a Gal4 activation domain, was transformed into AH109 cells. The TRBD of *G. duodenalis* TERT (808–1464 bp) was amplified by PCR (forward, 5′-GAA TTC ATT ACA AGT ACT AGA GTA GTA AAT T-3′; reverse, 5′-GGA TCC CGA TAG ACA AAC GAT AGC CTA C-3′) to construct an *Eco*RI-*Bam*HI fragment of *G. duodenalis* cDNA and ligated into pGBKT7 as the bait. The bait, which contains a Gal4 DNA-binding domain, was transformed into Y187. An α-galactosidase assay was performed to assess autoactivation.

The yeast two-hybrid screen was carried out in accordance with Matchmaker Library Construction and Screening Kit (Clontech). The clones were spread on SD/-His/-Leu/-Trp/3-AT and SD/-Ade/-His/-Leu/-Trp/X-α-gal selection media, and the appearance of blue colonies indicated a positive result. Positive (co-transformed with pGBKT7-53 and pGADT7-T) and negative (co-transformed with pGBKT7-Lam and pGADT7-T) controls were also included. The positive transformants were sequenced by Sangon Biotech (Shanghai, China).

### Expression, purification, and identification of the TRBD and ZFD

ZFD was cloned into pGEX-4T-1 vector to generate a GST tag. TRBD was ligated with 6X His-tag vector pET-32a (+), generating His-tagged proteins. For protein expression, *Escherichia coli* BL21(DE3) was grown in 200 mL LB-Amp media at 37 °C until an OD600 of 0.6–0.8 was reached. After induction with 0.25 mmol/l IPTG at 16 °C for another 20 h, the cells were harvested and lysed by sonication in chilled lysis buffer. Cell lysates contained His-TRBD or GST-ZFD were clarified three times by centrifugation at 30,000×*g* for 30 min. The supernatants were subjected to Ni-NTA affinity chromatography using a column pre-equilibrated with buffer (Qiagen, Chatsworth, CA) or glutathione-triethyleneglycocyl-sepharose 6B (CoWin Biotech, Beijing, China) according to instructions by manufacturers, respectively. Unbound proteins were removed with wash buffer, and target proteins were eluted with elution buffer. The final protein concentration was determined using a BCA kit (Thermo Fisher Scientific, Waltham, MA, USA).

### Pulldown assays

Pulldown experiments were performed as described previously [24]. His-TRBD was incubated with GST-ZFD in lysis buffer (10 mmol Tris-HCl (pH 7.5), 150 mmol NaCl, 1% NP-40, 1 mM EDTA, and complete protease inhibitor cocktail from Roche) at 4 °C overnight. The beads were washed with lysis buffer five times, and the bound proteins were analyzed by western blotting. The GST protein alone incubated with His-TRBD was used as a negative control. Moreover, the groups of the whole TERT and the C-terminal part (360 aa) of TERT in pulldown are also used as control groups. The intensity of each band was measured using ImageJ software.

### Co-immunoprecipitation and western blotting analyses

293T cells were transfected with the recombinant plasmids pcDNA3.1-Myc-TRBD/pcDNA3.1-HA-ZFD, pcDNA3.1-Myc-TRBD, and pcDNA3.1-HA-ZFD using Lipofectamine 2000 (Invitrogen, Waltham, MA, USA). The proteins were immunoprecipitated with anti-HA (Sigma-Aldrich, St Louis, MO, USA) or anti-Myc (Sigma-Aldrich) antibodies and protein A beads. The beads were washed three times with 1 ml of IP buffer, SDS sample buffer was added, the samples were boiled to elute the bound proteins, and the eluates were analyzed by WB.

### SNAP-tag protein complementation assay in 293T cells

To verify interaction between TRBD and ZFD, TRBD and ZFD were amplified by PCR and inserted into cSNAP and nSNAP, respectively, to generate pTRBD-cSNAP and pZFD-nSNAP plasmids in accordance with previously described methods. The PCR primers for ZFD were forward (5′-CTA GCT AGC TAG ATG AGC ATA GTG ATG CGC AAT A-3′) and reverse (5′-TCG CGA TCG CGA TTA CTT GGC TGT GGC TGC TCC G-3′) and for TRBD were forward (5′-CTA GCT AGC TAG GAC AAG AAA CCC CAA TCT CT-3′) and reverse (5′-CCG CTC GAG CGG CGA TAG ACA AAC GAT AGC CTA C-3′). Combinations of pTRBD-cSNAP and pZFD-nSNAP plasmids were co-transformed into 293T cells in accordance with previously described methods. GFP fluorescence was visualized and imaged by laser scanning confocal microscopy (FluoView FV1000, Olympus, Japan).

### Virus-mediated SNAP-tag protein complementation assay in *G. duodenalis*

To verify the PPI location in *G. duodenalis*, the TRBD-cSNAP and ZFD-nSNAP fragments were inserted into the pC631 viral vector. GFP fluorescence was visualized and imaged by laser scanning confocal microscopy (FluoView).

### Structure of the hammerhead ribozyme

Using RNA structure software, the full-length *Giardia lamblia* ATCC 50803 ZFD (GL50803_20802) was analyzed, and the secondary structure of the mRNA was predicted. According to the position of the GUC sequence and the nucleotide sequences flanking it, a triplet GUC located at position 257 of ZFD mRNA was chosen as the target site for the ribozyme. The designed hammerhead ribozyme consisted of 52 nucleotides, including a 23 nt catalytic core and 29 nt of flanking antisense sequences. The sequence was 5’-GTG TTT GGA AGC GCT GAT GAG TTC CGT GAG GAC GAA ACA AGC TCT TTA TGC T-3’ (the underlined nucleotides are the catalytic core). The primers used for the ribozyme were as follows: forward (5′-CGC GGA TCC GCG GTG TTT GGA AGC GCT GAT GAG TTC CGT GAG GAC GAA ACA AGC TCT TTA TGC TTC CCC GCG GGG A-3′ (*Bam*HI); reverse, 5′-TCC CCG CGG GGA AGC ATA AAG AGC TTG TTT CGT CCT CAC GGA ACT CAT CAG CGC TTC CAA ACA C-3′ (*Sac*II). After amplification by PCR, the double-stranded DNA was inserted into the pC631-SNAP vector to construct pC631-SNAP-Ham-ZFD. DH5α was transformed with the plasmid and plated on LB solid medium containing ampicillin. Positive colonies were sequenced by Sangon Biotech (Sangon Biotech, Shanghai, China).

### *In vitro* transcription

To construct pC631-SNAP-ZFD, ZFD was amplified by PCR: forward (5′-CGC GGA TCC GCG ATG AGC ATA GTG ATG CGC AAT A-3′) (*Bam*HI); reverse (5′-TCC CCG CGG GGA TTA CTT GGC TGT GGC TGC TCC G-3′) (*Sac*II), and the product was inserted into the pc631-SNAP vector. Both pc631-SNAP-ZFD and pC631-SNAP-Ham-ZFD were linearized by *Nru*I at the 3’-end. The two transcripts were each synthesized *in vitro* with RNA Production System (Promega, Madison, WI, USA).

### In vitro ribozyme cleavage of ZFD mRNA

For the *in vitro* ribozyme cleavage assay, pC631-SNAP-ZFD and pC631-SNAP-Ham-ZFD were mixed at a 1:5 molar ratio in Tris-HCl (50 mM, pH 8.0). The mixture was vortexed and maintained at 95 °C for 3 min, centrifuged briefly at room temperature and incubated at 37 °C for another 5 min. Finally, MgCl_2_ was added to start the cleavage reaction (final concentration, 10 mM), which was catalyzed at 37 °C for 3 h. The reaction product was then analyzed by RT-PCR.

### Electrotransformation

For the *in vivo* ribozyme cleavage assay, trophozoites of virus-free *G. duodenalis* mixed with pC631-SNAP-Ham-ZFD were electroporated at 2.5 kV, 25 mF, 400 fQ, and a 0.2 time constant (three times) using a 0.1-cm electrode gap cuvette. The cells were incubated for an additional 15 min on ice after electroporation. Control samples were treated similarly but without electroporation. Negative control samples were electroporated with pC631-SNAP vectors.

### Telomerase assay

The TRAP assay was performed as follows. First, cell extracts were added to the telomerase extension reactions and incubated for 20 min at 37 °C. PCR was performed using the following primers: forward (5′-AAT CCG TCG AGC AGA GTT-3′); reverse (5′-CAA CAT CTC CAC TAC CTT CCT ACC CTA C-3′). As an internal telomerase assay standard, internal primers were added to the PCR mixture as described previously. The telomerase products were resolved by 12% nondenaturing polyacrylamide gel electrophoresis. Bands were detected for densitometry analysis using BandScan software.

### qPCR test for telomere length measurement

qPCR was employed to measure TL using the method described by Cawthon. Briefly, TL primers (forward, 5′-GCT TTA GCC CAG CCC AGC CCA-3′; reverse, 5′-CTT TCG AGG AGG GGA GGG GA-3′) and a single gene primer (GenBank: L29032.1) (forward, 5′-TTA CCA CGA GCG CAG AGT TT-3′; reverse, 5′-TAG GCG AGA CCC CAC TTG TA-3′) were used. All qPCR assays were conducted using a Rotor-Gene Q (Qiagen) real-time PCR system with the following cycling parameters: 5 min at 95 °C, followed by 30 cycles of 7 s at 98 °C and 10 s at 58 °C. The TL for each sample was determined using the telomere to single-copy gene ratio (T/S ratio) by calculating the ΔCt [Ct(telomere)/Ct(single gene)]. The T/S ratio for each sample (x) was normalized to the mean T/S ratio of the reference sample [2^−(ΔCtx−ΔCtr) ^=  2^−ΔΔCt^], which was also applied for the standard curve, both as a reference sample and as a validation sample.

### Experimental design and statistical analysis

The *G. duodenalis* wildtype strain, yeast strains Y187, AH109 and 293T cell line are stored at Jilin University in China. At least three independent biological replicates of each treatment were carried out for statistical analyses. *P*-values were determined by one-way and two-tailed ANOVAs. The results are expressed as the mean ± standard error, SE. Asterisks indicate statistically significant correlations at the 95 % confidence level.

## Results

### ZFD is a novel TRBD-interacting protein

To identify proteins involved in regulating the TRBD, we screened a *G. duodenalis* cDNA library using the TRBD of *G. duodenalis* TERT (808–1464 bp) as bait in a yeast two-hybrid system. Three interacting clones were identified from 1.2 × 10^7^ transfectants. NCBI BLAST software showed that the clone sequence was identical to that of *Giardia lamblia* ATCC 50803 ZFD (GL50803_20802) (Additional file 1: Figure S1a, *n* = 3). Direct two-hybrid binding assay results confirmed the specificity of the interaction. In addition, an α-galactosidase assay indicated that TRBD did not activate the reporter gene, suggesting a specific interaction between TRBD and ZFD (Additional file 1: Figure S1b). pGBKT7-TRBD plasmid toxicity was also tested (Additional file 1: Figure S1c), and the results showed no significant difference between the empty vector and pGBKT7-TRBD.

**Fig. 1:**
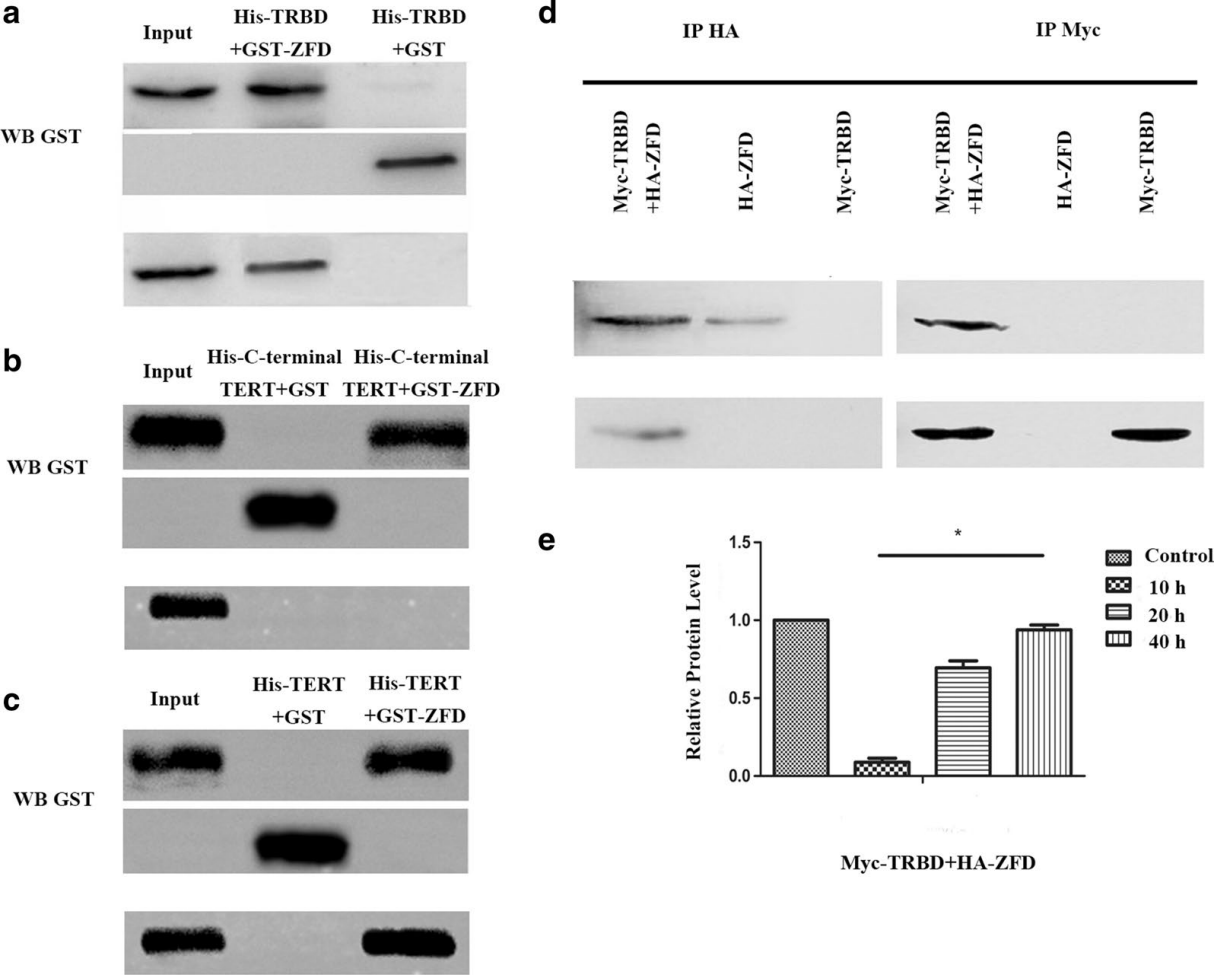
A pulldown assay and co-immunoprecipitation validate the interaction between TRBD and ZFD. **a** Binding of His-TRBD to GST-ZFD immobilized on glutathione beads was determined by WB analysis. Total protein was probed with an anti-His or anti-GST antibody as indicated. **b** Binding of His- C-terminal part of TERT to GST-ZFD immobilized on glutathione beads was determined by WB analysis. Total protein was probed with an anti-His or anti-GST antibody as indicated. **c** Binding of His-TERT to GST-ZFD immobilized on glutathione beads was determined by WB analysis. Total protein was probed with an anti-His or anti-GST antibody as indicated. **d** Interaction between TRBD and ZFD in 293T cells. Immunoprecipitation was detected by WB with anti-HA and anti-Myc antibodies. **e** TRBD-ZFD protein was detected at 10 h, 20 h, and 40 h. *P < 0.05

To demonstrate interaction between the TRBD and ZFD *in vitro*, TRBD, TERT, C-terminal part of TERT and ZFD were amplified and cloned into pET-32a and pGEX-4T-1 to construct pET-32a-TRBD, pET-32a-TERT, pET-32a-C-terminal part of TERT and pGEX-4T-1-ZFD expression plasmids, respectively. His-TRBD, His-TERT, His-C-terminal part of TERT and GST-ZFD proteins were purified from *E. coli* (Additional file 2: Figure S2), pulled down and analyzed by western blot (WB) assays with anti-His or anti-GST antibodies. As shown in Fig. 1a–c, the WB analysis indicated that His-TRBD bound directly to GST-ZFD *in vitro* but not to GST alone, which was consistent with the results of the two-hybrid analysis. To further confirm the interaction, co-IP was performed in 293T cells transfected with Myc-TRBD and/or HA-ZFD. When the cell lysates were immunoprecipitated with the anti-HA or anti-Myc antibody, co-precipitation was detected only with the Myc-TRBD/HA-ZFD group and not in the single plasmid or control groups. Moreover, the TRBD-ZFD expression level increased from 10 h to 40 h (Fig. 1d, e). These results suggest that the TRBD directly interacts with ZFD, which is consistent with the results of the pulldown assays. These findings indicate that ZFD is a novel binding protein of TRBD.

**Fig. 2:**
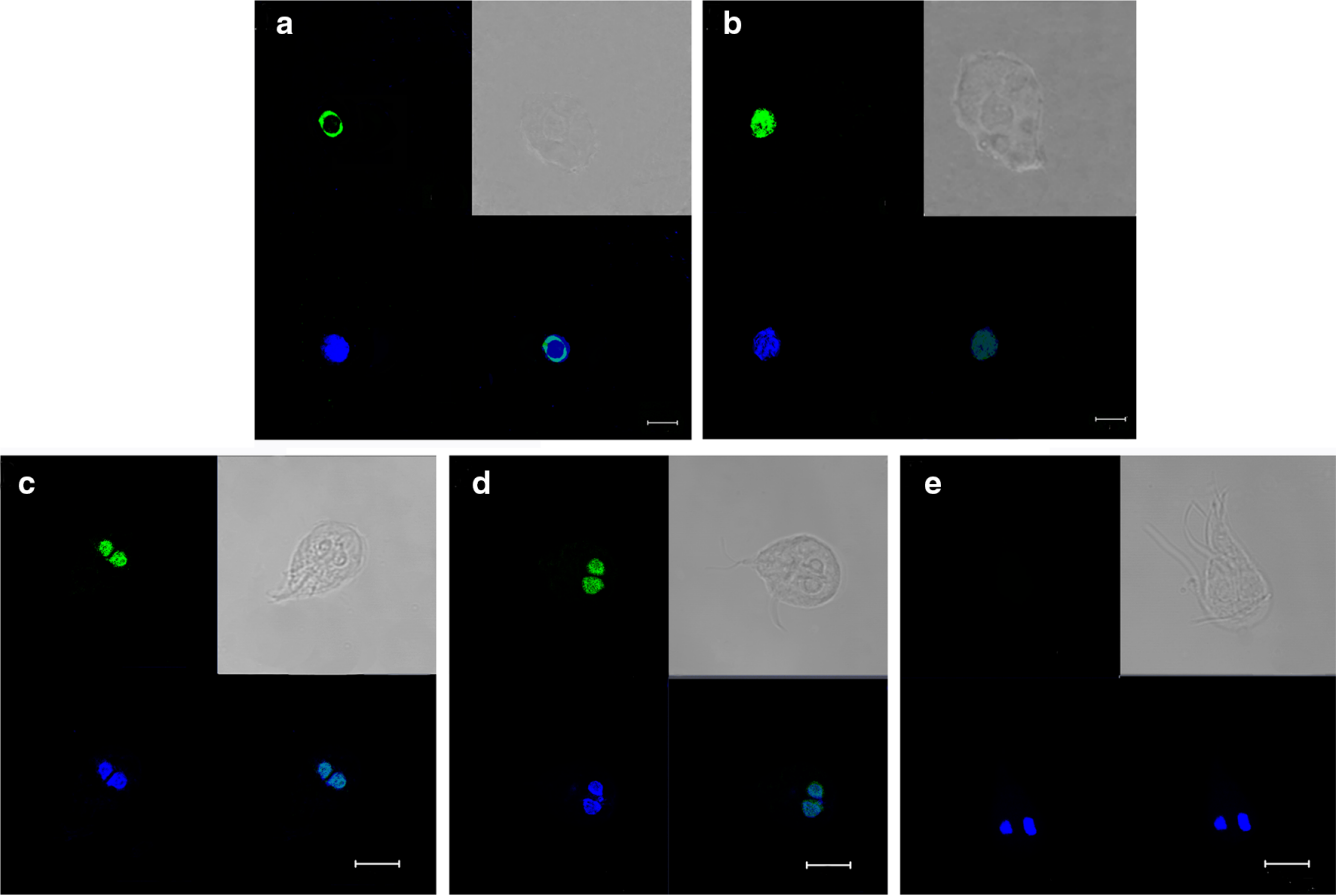
Interactions between the TRBD and ZFD in cells. Interactions between the TRBD and ZFD in 293T cells and *G. duodenalis*. **a** Co-infection of 293T cells with pcDNA-NLS-FRB-cSNAP and pcDNA-nSNAP-FKBP12. **b** Co-infection of 293T cells with pTRBD-cSNAP and pZFD-nSNAP. **c** Co-infection of *G. duodenalis* with pTRBD-cSNAP and pZFD-nSNAP. **d** Co-infection of *G. duodenalis* with pC631-Jun-cSNAP and pC631-Fos-nSNAP. **e** Negative control. *Scale-bars*: 5 µm

Interaction between the TRBD and ZFD in living cells was also investigated using the SNAP-tag protein complementation assay, which enables visualization of protein complex formation and the location of protein interactions in living cells. The TRBD was fused to cSNAP (pTRBD-cSNAP), and ZFD was fused to nSNAP (pZFD-nSNAP). Co-expression of pTRBD-cSNAP and pZFD-nSNAP bimolecular fluorescence complementation (BiFC) vectors in 293T cells resulted in fluorescence complementation, which was observed as green dots predominantly in the nucleus; in contrast, no fluorescence was detected in reactions containing pTRBD-cSNAP or pZFD-nSNAP alone. Fluorescence complementation between NLS-FRB and FKBP12 fused to cSNAP (pcDNA-NLS-FRB-cSNAP) and nSNAP (pcDNA-nSNAP-FKBP12), respectively, was used as a positive control (Fig. 2a, b). Subsequently, TRBD-cSNAP and ZFD-nSNAP fragments were inserted into the *Giardia* viral vector pC631 to form pC631-TRBD-cSNAP and pC631-ZFD-nSNAP, respectively. Fluorescence complementation between Jun and Fos fused to cSNAP (pC631-Jun-cSNAP) and nSNAP (pC631-nSNAP-Fos), respectively, was used as the positive control. The results showed that PPIs occurred in both nuclei of *G. duodenalis* (Fig. 2c–e).

### Reduction in ZFD inhibits the proliferation of *G. duodenalis*

A hammerhead ribozyme targeting ZFD was designed to knock down ZFD expression in cells. Using a computer-predicted RNA secondary structure, we selected the triplex GUC of the TERT mRNA as the cleavage site and then constructed the hammerhead ribozyme (Ham-ZFD), which included a ZFD antisense sequence and catalytic core. Ham-ZFD was inserted into the viral expression vector pC631-SNAP, which contains the 631 nt 5′-end and the 2022 nt 3′-end of the *Giardia* viral genome cDNA and T7 promoter. After transcription *in vitro*, the RNA concentrations of pC631-SNAP-Ham-ZFD, pC631-SNAP-ZFD, pC631-SNAP-Ham-GDH and pC631-SNAP-ZFD-Sub were 11.2 μg/µl, 11.5 μg/µl, 10.8 μg/µl and 10.7 μg/µl, respectively.

**Fig. 3:**
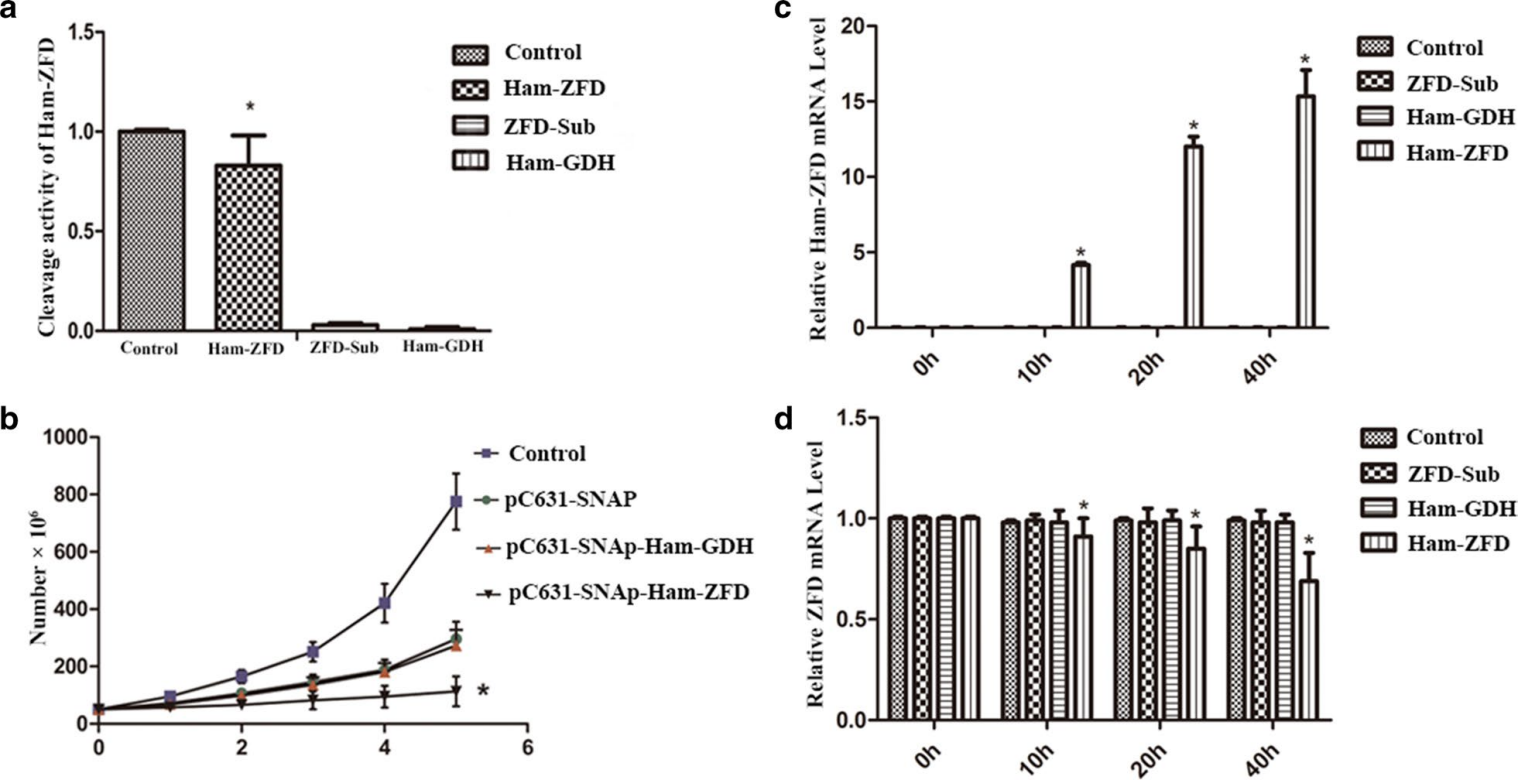
The effect of Ham-ZFD in *G. duodenalis* cell lines. **a** Cleavage activity of the hammerhead ribozyme *in vitro*. *P < 0.05. **b** Trophozoite count. Control: trophozoites without electroporation; pC631-SNAP: trophozoites electroporated with a plasmid without the ribozyme; pC631-SNAP-Ham-GDH: trophozoites electroporated with a plasmid harboring the Ham-GDH ribozyme; pC631-SNAP-Ham-ZFD: trophozoites electroporated with a plasmid harboring the Ham-ZFD ribozyme. *P < 0.05. **c** Relative Ham-ZFD mRNA levels in Ham-ZFD-transfected *G. duodenalis* cell lines. **d** Relative ZFD mRNA levels in Ham-ZFD-transfected *G. duodenalis* cell lines. *P < 0.05

pC631-SNAP-ZFD was mixed with pC631-SNAP-Ham-ZFD, pC631-SNAP-Ham-GDH and pC631-SNAP-ZFD-Sub, and the remainder of the pC631-SNAP-ZFD RNA was reverse transcribed into cDNA and analyzed by real-time PCR. The results confirmed 84% knockdown of ZFD, whereas less than 3% of ZFD was cleaved in the negative control (Ham-GDH) and Sub-ZFD (only ZFD antisense sequence) groups (Fig. 3a, Additional file 3: Figure S3).

To confirm the results *in vivo*, we knocked down ZFD expression in *G. duodenalis* by transfecting ZFD-targeting ribozymes. Because pC631-SNAP-Ham-ZFD contained *Giardia* viral genome cDNA, green fluorescence was visible in the whole trophozoite. The Ham-ZFD proliferation rate was similar to that of wildtype cells in the first two days, but the cell density on the 5th day was only approximately 41% of that of wildtype cells (Fig. 3b). Moreover, the presence of Ham-ZFD and decrease in ZFD were monitored by RT-PCR (Fig. 3c, d). These data suggest that electroporation with Ham-ZFD in *G. duodenalis* may reduce cell growth.

### Reduction in ZFD decreases telomere length in *G. duodenalis*

**Fig. 4:**
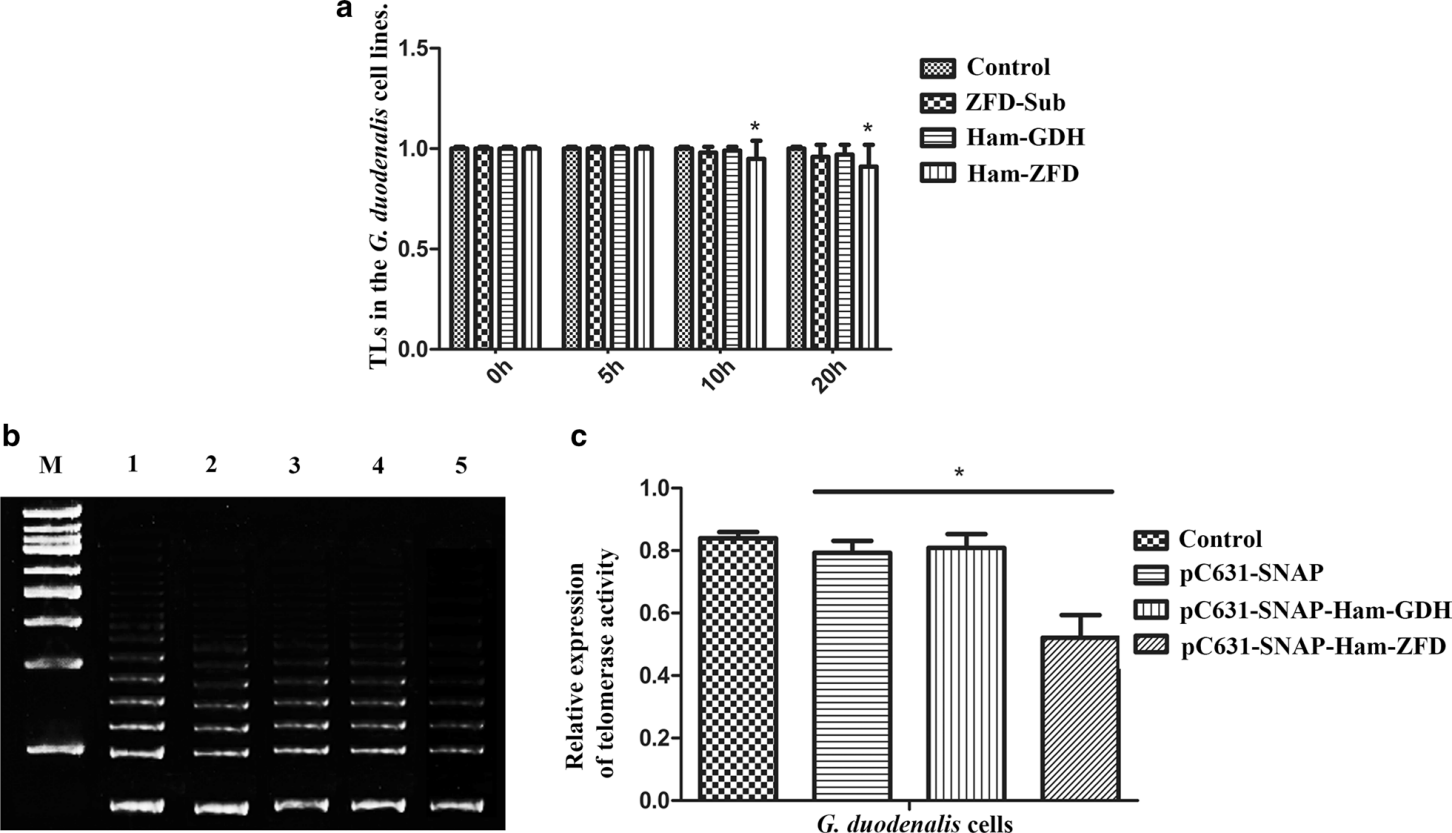
TLs and Telomerase activity in ribozyme Ham-ZFD-transfected *G. duodenalis* cell lines. **a** TLs in ribozyme Ham-ZFD-transfected *G. duodenalis* cell lines. Control: non-transfected *G. duodenalis* cell lines; ZFD-Sub: *G. duodenalis* with the pC631-SNAP-ZFD-Sub plasmid. Ham-GDH: *G. duodenalis* with the pC631-SNAP-Ham-GDH plasmid; Ham-ZFD: *G. duodenalis* with the pC631-SNAP-Ham-ZFD plasmid; *P < 0.05. **b** Telomerase activity in *G. duodenalis* cell lines. M: DL500 DNA marker; 1: TRAP products from HeLa cells; 2: TRAP products from untreated *G. duodenalis* cells; 3: TRAP products from *G. duodenalis* cells with pC631-SNAP; 4: TRAP products from *G. duodenalis* cells with pC631-SNAP-Ham-GDH; 5: TRAP products from *G. duodenalis* cells with pC631-SNAP-Ham-ZFD. **c** Telomerase activity in *G. duodenalis* cell lines. *P < 0.05. Control: *G. duodenalis* without pC631-SNAP plasmid; SNAP: *G. duodenalis* with pC631-SNAP plasmid; Ham-GDH: *G. duodenalis* with pC631-SNAP-Ham-GDH plasmid; Ham-ZFD: *G. duodenalis* with pC631-SNAP-Ham-ZFD plasmid; *P < 0.05

qPCR analysis of *G. duodenalis* was employed to measure telomere length (TL) as previously described with the formula $\left[ {{{ 2^{{{\text{C}}({\text{telomeres}})}}_{\text{t}} } \mathord{\left/ {\vphantom {{ 2^{{{\text{C}}({\text{telomeres}})}}_{\text{t}} } { 2^{{C({\text{single}})}}_{\text{t}} }}} \right. \kern-0pt} { 2^{{C({\text{single}})}}_{\text{t}} }}} \right]^{ - 1} = 2^{{{-}\Delta C}}_{\text{t}}$. The actin gene (GenBank: L29032.1) was used as a single-copy gene control. The relative telomere to single-copy gene ratio (T/S) ratio (T/S of one sample relative to the T/S of another sample) was calculated using the formula $2^{{{-}(\Delta C 2}}_{\text{t}} -^{{{-}\Delta C 1)}}_{\text{t}} = 2^{{{-}\Delta \Delta C}}_{\text{t}}$. To verify the effect of the hammerhead ribozyme, the TLs in the Ham-ZDF, ZFD-Sub and Ham-GDH were analyzed. The TL in the Ham-ZFD group was 0.91 ± 0.07 relative to that in the control group TL after 20 h. The TLs of the Sub-ZFD and Ham-GDH groups were 0.97 ± 0.05 and 0.96 ± 0.02 relative to the control group TL, respectively (Fig. 4a). These results suggest that the hammerhead ribozyme does not affect the TL and ZFD may be partly involved in TL control.

### Reduction in ZFD decreases telomerase activity in *G. duodenalis*

A TRAP assay was performed to reveal whether ZFD affects telomerase activity in *G. duodenalis*. A ladder of typical TRAP products was observed in all of the groups, with the Ham-GDH and Sub-ZFD groups not differing from the control group. However, loss of telomerase activity was observed in the Ham-ZFD group (Fig. 4b, c), indicating that the decrease in ZFD reduced telomerase activity in *G. duodenalis*. In addition, telomerase activity in the Ham-ZFD group was only approximately 62% of that in the control group.

## Discussion

Since Muller [25] discovered special sequences at the end of chromosomes and named them telomeres, telomeres, telomerase, and telomerase-related proteins have become hot research topics [26]. Telomere assembly, activation and attrition are complex processes driven by telomerase that show similarities and differences in various organisms [27]. Studying telomerase is helpful for understanding the mechanisms involved and providing research directions for cell life-cycle control. Telomerase complexes generally contain TERT, TER, and proteins that bind to RNA or are involved in the stabilization and maturation of telomerase complexes.

The TERT component was first identified in *Euplotes aediculatus* and later in numerous eukaryotes, including humans, mice, *Saccharomyces cerevisiae*, *Oxytricha trifallax*, *Leishmania amazonensis* and *Trichinella spiralis* [28–31]. Regarding parasites, the gene encoding TERT (2385 nt) was detected from *Plasmodium falciparum* in 2005, and remarkable sequence diversity has been observed in different *Plasmodium* species [18, 32]. The TERT gene sequence of *Leishmania major* was used to clone the same gene from other parasite species identified by Miriam [33] (*L. amazonensis*, *L. braziliensis* and *L. donovani*). Miriam [33] also showed that the TERT catalytic subunit is a universally conserved feature of telomerases in these species. In 2004, Xu et al. [34] reported the TERT gene in *C. parvum* (4407 nt) but did not conduct relevant mechanistic research.

TERT regulation at the transcriptional level is considered the main mechanism affecting telomerase activity, but some studies have noted that PPIs and protein-level regulation also control telomerase [35, 36]. PPIs are important for various life processes, such as signal transduction, gene expression regulation, energy and material metabolism, and cell cycle regulation [37]. Studying PPIs in biological systems is key for understanding protein mechanisms, responses to biological signals and energy metabolism under special physiological conditions such as disease, and functional links between proteins [38].

Because telomerase characteristics differ among species, the function of telomeric DNA-binding proteins in telomerase and telomere regulation also differ. In ciliates, yeasts and vertebrates, telomerase-related proteins have been shown to play important roles in regulating telomerase assembly, posttranslational modification, localization, and enzyme function [39, 40]. The protein p80 in mammalian cells is related to telomerase activity *in vivo* [41]. MKRN1 is a well-known telomerase modifier in the ubiquitin-proteasome pathway, and it promotes hTERT degradation, leading to a reduction in telomerase activity and TL [42]. The hTERT protein level reportedly increases significantly after gene silencing of PCDH10, indicating that PCDH10 likely affects hTERT protein stability *via* PPIs and thus regulates telomerase activity [43]. In yeast, Ku binds specifically to the TER neck-ring and promotes telomerase aggregation at telomere ends [44]. In parasite research, extraction of telomeres from *T. thermophila* has revealed p80 and p95 to be as telomerase-related proteins, and the data suggest that p80 and p95 have good affinity for telomerase [45]. Moreover, Zhao et al. [24] found that TERT-related proteins in *Eimeria tenella* can interact with 14-3-3 proteins and negatively regulate telomerase activity. These results suggest that TERT may play an important role in regulating telomerase activation *via* PPIs. However, research into the relevant mechanism is lacking.

*Giardia duodenalis* is a model organism that presents immortal proliferation mainly due to telomerase complex-induced regulation. Because the molecular biological characteristics of *Giardia* are poorly understood, few ideal vaccines can be used in the clinic [15–19]. Although GlTERT (2883 nt), a repeat sequence (TAGGG)n and a retrotransposable element in the *G. duodenalis* genome were identified in 2000, only one study on *G. duodenalis* telomerase has been conducted to date [46]. To investigate TERT/telomerase-related proteins in *G. duodenalis*, we first constructed a cDNA library for *G. duodenalis* and screened it using the yeast double-hybrid method to identify a related zinc-finger protein, ZFD (GL50803-20802), interacting with the TRBD. The screening results were verified using co-IP and pulldown experiments.

Zinc fingers proteins were first identified in a study of transcription in the African clawed frog *Xenopus laevis* [37], and they are now known to be widely distributed in animals, plants, and microorganisms [38, 47, 48]. Indeed, nearly 1% of the human genome may encode proteins containing zinc-finger structures [49]. Many zinc-finger proteins are transcription factors that play an important role in gene regulation. ZFD-containing proteins are involved in regulating cell replication, repair, transcription, translation, metabolism, signal conduction, cell proliferation and apoptosis through specific binding to target molecular DNA, RNA, and proteins [50, 51]. Thus far, the study of the zinc finger proteins of *G. duodenalis* has primarily focused on variant-specific surface proteins (VSPs), and an oral vaccine against *G. duodenalis* was designed to target VSPs [52]. However, the protein sequence obtained in this study does not belong to the VSP family because no CRGKA motif exists at the C-terminus. Notably, the protein sequences obtained in *G. duodenalis* are less than 6% homologous to those of other species, suggesting that ZFD may have a special role in *G. duodenalis*.

The most obvious feature of *G. duodenalis* is the two morphologically identical nuclei, though whether these two nuclei are indeed identical is unknown, as are the locations of the TRBD and telomerase-interacting proteins. Protein-fragment complementation assays (PCAs) were applied to determine the site of interaction between ZFD and the TRBD in both mammalian cells and *G. duodenalis*, and BiFC PCAs have been widely used to observe PPIs in living cells [53–55]. This assay was first described by Regan, who used two proteins of interest fused to the N-terminal or C-terminal non-fluorescent fragment of a fluorescent protein; the proteins were then co-expressed in cells [56]. Using fluorescence microscopy, an interaction occurring between the two proteins of interest is visualized by a fluorescent signal. To date, YFP and GFP have been used in BiFC assays in yeast, plant and mammalian cells and in drug discovery related to a specific PPI [57–59]. However, few reports have used the BiFC assay in *G. duodenalis* because GFP must be oxygenated for maturation and to become fluorescent. To solve this problem, we used the SNAP-tag protein complementation assay to visualize PPIs in living cells, as previously reported [58, 60].

We first utilized the split SNAP-tag labeling method and fused ZFD and TRBD to cSNAP and nSNAP, respectively and then inserted them into a mammalian plasmid. The results suggested that the interaction between ZFD and TRBD occurs in the nucleus of 293T cells. ZFD and TRBD were also fused to cSNAP and nSNAP, respectively, and inserted into the *Giardia* viral vector pC631, and the results confirmed that PPIs consistently occurred in both nuclei. Construction of a new virus-mediated split SNAP-tag labeling plasmid offers a new tool for imaging PPI events in *G. duodenalis* and may provide a new method of discovering PPIs in other anaerobic organisms *via* virus-mediated plasmids.

TERT variants influence TL and telomerase activity, though the correlation between TL and telomerase activity is unclear. Some studies have shown a lack of correlation between TL and telomerase activity and expression in leukemic cells [56]. Others have shown that the mean telomere length (TRF) in peripheral blood leukocytes correlates negatively with telomerase activity in aplastic anemia patients [60]. Moreover, modified TERT transiently enhances telomerase activity and rapidly extends telomeres [61]. To determine the relationship between ZFD and TL and telomerase activity in *G. duodenalis,* we constructed a ZFD ribozyme and found that a reduction in ZFD mRNA led to a decreased reproduction rate and a lower number of *G. duodenalis*. Telomerase activity and TL measurements revealed that reducing the ZFD mRNA level can decrease telomerase activity and shorten the TL. Because the mechanism by which ZFD regulates TL and telomerase activity remains not well understood, we will conduct further systematic studies of ZFD with regard to cell activity and other aspects of telomeres in future research.

## Conclusions

In this study, we used the yeast double-hybrid assay to identify a new type of ZFD that interacts with the TRBD and examined the interaction using co-IP and pulldown assays. We then used split SNAP-tag labeling to determine the site of the PPI in 293T cells. To ensure that split SNAP-tag labeling can be used in *G. duodenalis*, a new type of virus-mediated split SNAP-tag labeling plasmid was constructed. Our results showed that PPIs consistently occurred in both *G. duodenalis* nuclei. Moreover, we constructed a ZFD ribozyme and found that decreases in ZFD mRNA reduced the reproduction rate, the number of *G. duodenalis* and telomerase activity and shortened the TL.

## Supplementary information

**Additional file 1: Figure S1.** Yeast screening results for the mating reaction between pGBKT7-TRBD-transformed Y187 and AH109. **a** Screening results for Y187 and AH109. Positive control: yeast screening positive control; negative control: yeast screening negative control; ZFD: zinc finger domain. **b** Autoactivation detection of pGBKT7-TRBD. Positive control: positive control plasmid pCL; bait plasmid: bait plasmid pGBKT7-TRBD. **c** Toxicity test for pGBKT7-TRBD. Positive control: empty vector; TRBD plasmid: pGBKT7-TRBD.

**Additional file 2: Figure S2.** The expression of recombinant proteins on a protein gel. **a** The SDS-PAGE analysis of purified C-terminal part protein. **b** The SDS-PAGE analysis of purified His-TRBD protein. **c** The SDS-PAGE analysis of purified GST-ZFD protein. **d** The SDS-PAGE analysis of purified whole TERT protein.

**Additional file 3: Figure S3.** Cleavage activity of the hammerhead ribozyme *in vitro*. **a** Standard curve for pC631-SNAP-Ham-ZFD. **b** Melting curve for pC631-SNAP-Ham-ZFD.

## Data Availability

All data generated or analyzed during this study are included in this published article.

## Abbreviations

CTE: c-terminal extension domain
ER: endoplasmic reticulum
PPI: protein–protein interaction
PVs: peripheral vesicles
RT: reverse transcriptase domain
TER: telomerase RNA
TERT: telomerase reverse transcriptase
TRBD: telomerase RNA-binding domain
ZFD: zinc finger domain
