# Supplementary figures and images for: Identification of a TRBD zinc finger-interacting protein in Giardia duodenalis and its regulation of telomerase
Source: Parasit Vectors. 2019 Nov 29;12:568. doi: 10.1186/s13071-019-3821-0 (PMC6884763; doi:10.1186/s13071-019-3821-0)

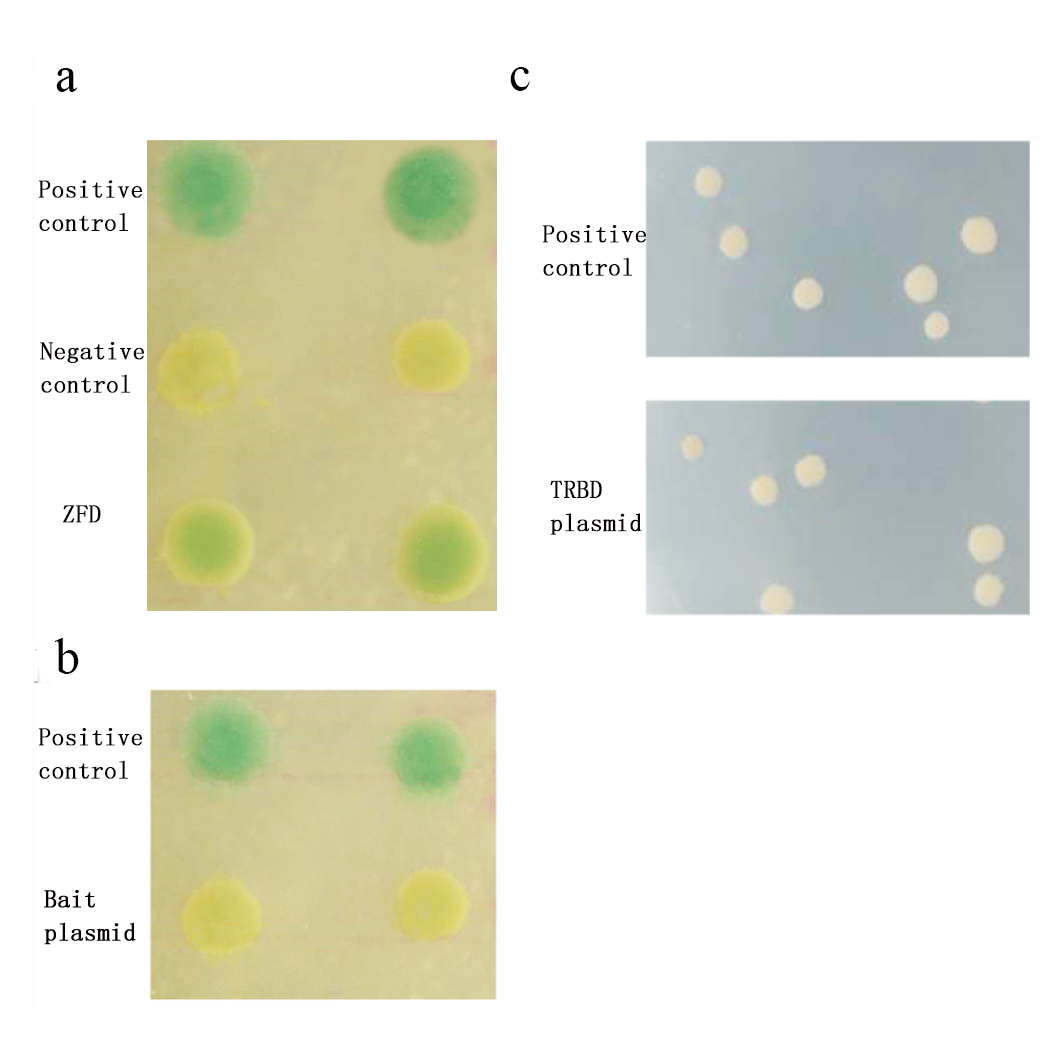

Supplement: Supplementary file 1 — Additional file 1: Figure S1. Yeast screening results for the mating reaction between pGBKT7-TRBD-transformed Y187 and AH109. a Screening results for Y187 and AH109. Positive control: yeast screening positive control; negative control: yeast screening negative control; ZFD: zinc finger domain. b Autoactivation detection of pGBKT7-TRBD. Positive control: positive control plasmid pCL; bait plasmid: bait plasmid pGBKT7-TRBD. c Toxicity test for pGBKT7-TRBD. Positive control: empty vector; TRBD plasmid: pGBKT7-TRBD. [file 13071_2019_3821_MOESM1_ESM.tif]

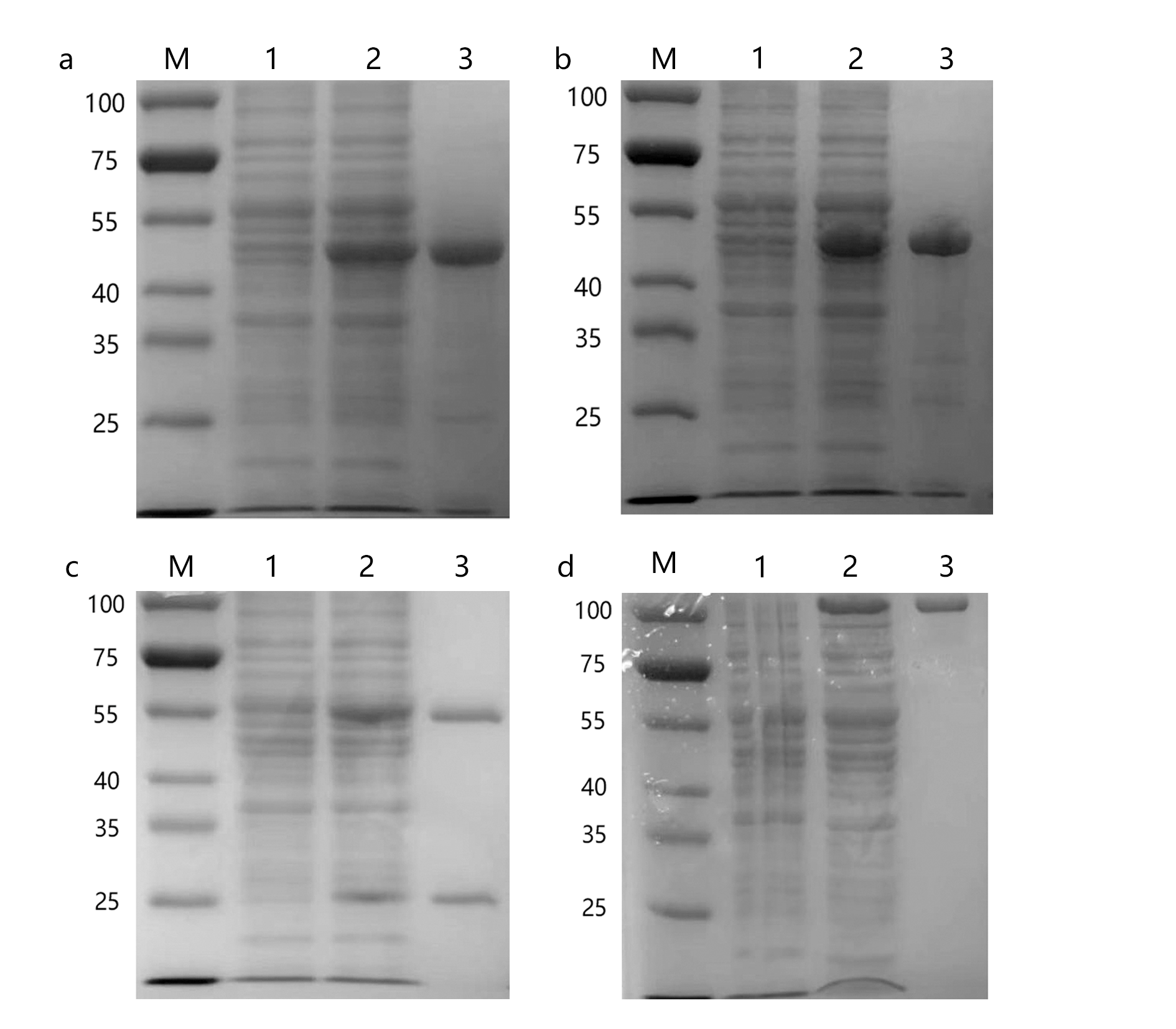

Supplement: Supplementary file 2 — Additional file 2: Figure S2. The expression of recombinant proteins on a protein gel. a The SDS-PAGE analysis of purified C-terminal part protein. b The SDS-PAGE analysis of purified His-TRBD protein. c The SDS-PAGE analysis of purified GST-ZFD protein. d The SDS-PAGE analysis of purified whole TERT protein. [file 13071_2019_3821_MOESM2_ESM.tif]

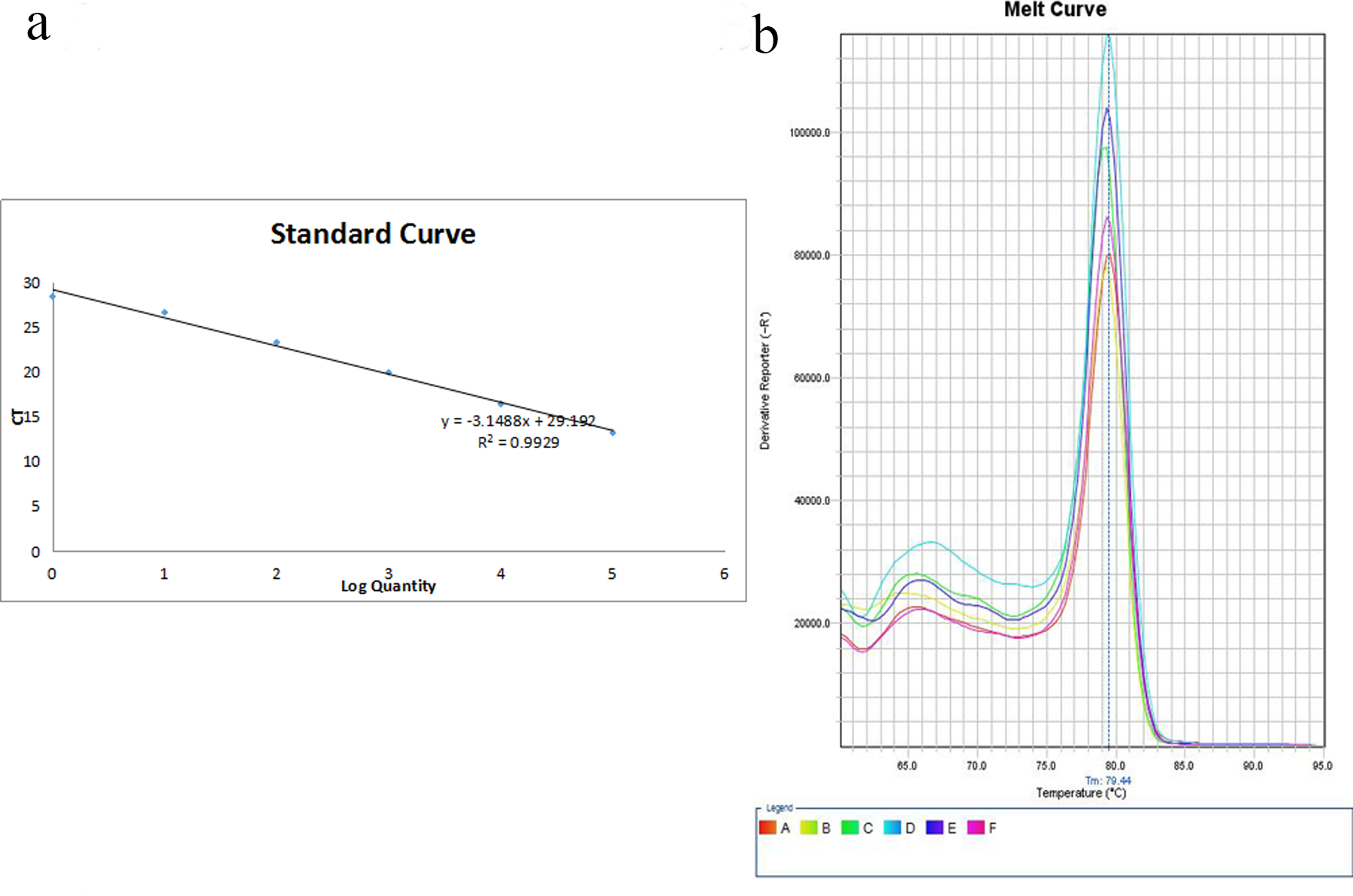

Supplement: Supplementary file 3 — Additional file 3: Figure S3. Cleavage activity of the hammerhead ribozyme in vitro. a Standard curve for pC631-SNAP-Ham-ZFD. b Melting curve for pC631-SNAP-Ham-ZFD. [file 13071_2019_3821_MOESM3_ESM.tif]
